# PERSONAL FACTORS ASSOCIATED WITH DROPOUT RATE IN SHORT-TERM FALL PREVENTION PROGRAM FOR OLDER ADULTS

**DOI:** 10.1093/geroni/igz038.449

**Published:** 2019-11-08

**Authors:** Qiwei Li, Becky P Knight

**Affiliations:** 1 University of North Texas, Denton, United States; 2 University of North Texas, Denton, Texas, United States

## Abstract

Fall prevention programs towards older adults are beneficial, however, drop-out rate among older adults in short-term fall prevention programs are relatively high (one out of three). With older adults dropping out, the programs benefit less for their target populations and achieve a lower level of cost-effectiveness as reported to funders. Literature review has revealed that previous studies on attrition frequently focus on longitudinal research, leaving the factors causing older adults to quit short-term (e.g., 8 weeks) fall prevention programs understudied. This study aimed to explore the association between demographic factors, educational level, health status, fall history, and the completion of the short-term fall prevention program named A Matter of Balance. A total of 691 older adults (526 females, 76.1%) 65 and older participated in the program were included and a Descriptive Discrimination Analysis (DDA) was conducted to analyze the multivariate relationship stated above. The results have identified that fall frequency, educational level, fear of falls, health status, perceived limitations due to fall, injury cases resulted from falls, and having arthritis are the most statistically significant factors that are associated with the completions among older adults for the short-term fall prevention program. The implications of this study include suggestions to modify future recruitment eligibilities in order to include the most suitable participants in certain programs, as well as adaptive modifications for short-term fall prevention programs that aim to serve larger aging populations with individualized conditions. Networking of evidence-based research outcomes should be shared for increased success of future programs.

